# Impact of Mineralocorticoid Receptor Antagonists in the Treatment of Heart Failure: Targeting the Heart Failure Cascade

**DOI:** 10.7759/cureus.45241

**Published:** 2023-09-14

**Authors:** Uday Jadhav, Tiny Nair, Padhinhare Mohanan, Vijay Chopra, Prafulla Kerkar, Arup Das Biswas, Prakash K Hazra, Nitin Zalte, Amarnath Sugumaran, Senthilnathan Mohanasundaram

**Affiliations:** 1 Department of Cardiology, Mahatma Gandhi Mission (MGM) New Bombay Hospital, Navi Mumbai, IND; 2 Department of Cardiology, PRS Hospital, Trivandrum, IND; 3 Department of Cardiology, Westfort Hi-Tech Hospital, Thrissur, IND; 4 Department of Cardiology, Max Super Speciality Hospital, New Delhi, IND; 5 Department of Cardiology, Asian Heart Institute, Mumbai, IND; 6 Department of Cardiology, Institute of Post-graduate Medical Education and Research and Seth Sukhlal Karnani Memorial (IPGMER-SSKM) Hospital, Kolkata, IND; 7 Department of Cardiology, AMRI Hospitals Limited, Kolkata, IND; 8 Medical Affairs, Cipla Ltd., Mumbai, IND

**Keywords:** mineralocorticoid receptor antagonists, heart failure, spironolactone, eplerenone, mra

## Abstract

Epidemiological data from the Indian subcontinent on the burden of Heart failure (HF) is scarce. Mineralocorticoid receptor antagonists (MRAs) are usually used in the management of HF and hypertension. A consortium of experts reviewed and opined on the pathophysiological role of aldosterone in HF and its cascading effects on the heart in terms of cardiac fibrosis, cardiac hypertrophy, and remodeling, increased propensity to cause arrhythmias in addition to its effect on sodium and water retention. This expert opinion document highlights the various mechanisms of action of MRAs. It provides clinical experience and practice-based expert opinion on the use of spironolactone and eplerenone in patients with HF. The role of MRAs in diabetic patients with HF has also been profiled.

## Introduction and background

Heart failure (HF) is affecting 26 million people worldwide and it is responsible for 1.8 million hospitalizations annually in India. The high prevalence of HF in India is proportionate to the increased presence of risk factors such as coronary artery disease, diabetes, hypertension, and chronic kidney disease on one side and diseases like rheumatic heart disease on the other side making HF “a dual burden” [1].

Epidemiological data from the Indian subcontinent on the burden of HF is scarce. Still, the estimate of HF incidence in India is around 1.3 to 23 million [2,3]. The prevalence of HF increases with age, and the lifetime risk of HF at 55 years is estimated to be 33% and 28.5% for men and women, respectively [3]. The exponential increase in the incidence of hypertension and diabetes mellitus contributes to the growing burden of HF seen today [3].

The chief pathogenic feature of HF is the inability of the heart to pump blood commensurate with the body’s requirement and need, secondary to either a structural or functional abnormality. Neurohormonal activation is an important compensatory mechanism that aids in the maintenance of mean arterial pressure in HF. When the mean arterial pressure falls, the renin-angiotensin-aldosterone system is activated, resulting in the conversion of angiotensinogen to angiotensin I and then to angiotensin II by the angiotensin-converting enzyme (ACE) released from the lungs. Angiotensin II directly increases vasoconstriction and stimulates the release of aldosterone, which causes water and sodium retention. This leads to further deterioration in the condition of the patient, who presents with congestion and dyspnea [4].

Mineralocorticoid receptor antagonists (MRAs) are a unique class of drugs that oppose/block the action of aldosterone and its harmful effects on the renal and extra renal system. They are usually used in the management of HF and hypertension. However, remained underutilized in clinical settings [5]. The objective of this clinical experience and practice-based expert opinion document is to guide physicians on the optimal use of MRAs in HF patients.

## Review

Mineralocorticoid receptor in heart failure

**Figure 1:**
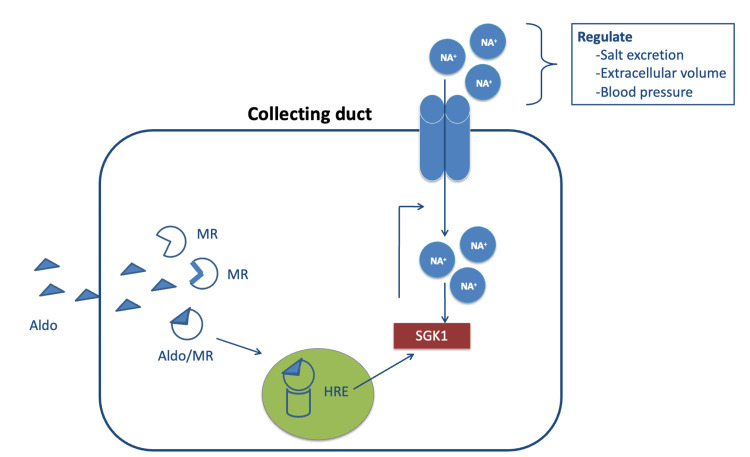
Effects of aldosterone mineralocorticoid receptor complex on collecting duct in the kidney Aldo, aldosterone; HRE, hormone response element; MR, mineralocorticoid receptor; SGK1, serum-inducible and glucocorticoid-inducible kinase 1 Reference no. [6]

Aldosterone is a mineralocorticoid hormone produced majorly by the adrenal cortex in response to angiotensin II release, hyperkalemia, and corticotropin [6]. Aldosterone binds to the mineralocorticoid receptor (MR) of the collecting duct of the kidney and stimulates serum-inducible and glucocorticoid-inducible kinase 1 (SGK1) gene expression and activates the epithelial sodium channel (ENaC) to regulate salt excretion, extracellular volume, and blood pressure (Figure 1) [6].

In addition to the epithelial effects, aldosterone has multiple negative non-epithelial effects on cardiomyocytes, endothelial cells, vascular smooth muscle cells (VSMCs), mesangial cells, and podocytes leading to induction of inflammation, stiffening of blood vessels, collagen formation, myocardial necrosis and stimulation of fibrosis [6,7].

Aldosterone plays an important role in heart failure pathogenesis. An elevated level of aldosterone tends to promote myocardial hypertrophy and remodeling, induction of fibrosis and apoptosis, contribute to endothelial dysfunction, and reduce myocardial perfusion, which results in increased incidence of cardiovascular events [6,7].

Aldosterone and its effects on sodium and fluid retention result in an undesired impact on fluid balance in patients with congestive HF, causing hypervolemia and promoting congestion. Furthermore, conditions like hypertension, atrial fibrillation, myocardial infarction, and diastolic HF increase the cardiac expression of MR. Therefore, increased MR activation levels could be observed even with normal levels of aldosterone and glucocorticoids [8].

MRA-mechanism of action

MRAs directly bind to and block MR, inhibiting aldosterone or 11-deoxycorticosterone from activating it, which reduces the degree of inflammation and remodeling in the heart. MRAs are classified as specific or non-specific based on their chemical class. Non-specific MRAs include spironolactone and specific MRAs include eplerenone [9]. MRAs are powerful treatment agents that have a marked beneficial effect on HF progression and resistant hypertension [10].

Effect of spironolactone and eplerenone on myocardial fibrosis and cardiac extracellular matrix turnover in heart failure

A sub-analysis of the randomized aldactone evaluation study (RALES) trial assessed the interactions between serum markers of cardiac fibrosis and the effect of spironolactone on outcomes in heart failure with reduced ejection fraction (HFrEF) and New York Heart Association (NYHA) Class III-IV [11]. In this trial, adding spironolactone (12.5 mg up to 50 mg) significantly decreased the levels of these serum markers of cardiac fibrosis. A significant reduction in the levels of the markers of cardiac extracellular matrix (ECM) turnover, namely procollagen type I amino-terminal peptide (PINP) and procollagen type III aminoterminal peptide (PIIINP) observed. The reduction in levels of PIIINP was more pronounced in HFrEF patients on spironolactone. The subgroup of patients with high baseline levels of PIIINP had a higher risk of mortality and this was significantly reduced after treatment with spironolactone. The study concluded that reduction of the excessive cardiac ECM turnover could perhaps be one of the extrarenal mechanisms contributing to the beneficial effect of spironolactone in patients with HFrEF NYHA Class III-IV [11].

The EPHESUS trial sub-analysis evaluated the effect of eplerenone in HFrEF patients post-acute myocardial infarction (AMI) with left ventricular systolic dysfunction (LVSD) by assessing the biomarkers of cardiac extracellular matrix turnover (PINP and PIIINP). The study demonstrated a significant reduction in PINP and PIIINP after treatment with eplerenone at six and nine months [12]. Insights from the EPHESUS and REMINDER trials stated that female sex, eplerenone, reperfusion therapy, serum K+ <4 mmol/L, circulating levels of PIIINP ≥ 3.6 ng/mL, and PINP ≥ 27 ng/mL were associated with a significant PIIINP decrease. A better response (decrease in PIIINP) to eplerenone was observed in patients with baseline PIIINP ≥ 3.6 ng/mL [13].

Effect of MRA on neurohormonal markers in heart failure

In RALES neuro-hormonal sub-group analysis a total of 107 patients with NYHA class III-IV (with a mean ejection fraction of 25%) were randomized to treatment with either spironolactone (25mg) or placebo. In the spironolactone-treated patients, the brain natriuretic peptide (BNP) plasma concentrations were reduced by 23% compared to the placebo (P<0.05) at six months. This reduction in natriuretic peptides is attributed to changes in left ventricular diastolic filling pressure and/or compliance [14].

In the TOPCAT (treatment of preserved cardiac function heart failure with an aldosterone antagonist trial) Bio-repository study (n=247 HFpEF patients), a significant reduction in the BNP levels from the baseline was observed in the spironolactone-treated group (122 ng/L to 102 ng/L) as compared to the placebo-treated group (118 ng/L to 137 ng/L); P=0.003. Moreover, a significant reduction in NT-proBNP in the spironolactone group (from 626 ng/L to 579 ng/L) compared to placebo (from 614 ng/L to 707 ng/L), P=0.044. Stating that the treatment with spironolactone in HFpEF patients has the potential effect of reducing cardiac and renal biomarkers after 12 months [15].

MRA in HFrEF

The RALES study demonstrated that the HFrEF patients (left ventricular ejection fraction (LVEF) 35%) on 25 mg spironolactone once daily had a 30% reduction in the risk of death from all causes, a 32% reduction in the risk of death from cardiovascular causes, 35% lower frequency of hospitalization for worsening HF, and a 30% reduction in the risk of hospitalizations from cardiac causes compared to placebo (P<0.001) [16].

The real-world study OPTIMIZE-HF evaluated spironolactone in elderly patients HFrEF and demonstrated that spironolactone use reduced all-cause mortality by 8% (P=0.13) and HF readmissions by 13% (P=0.04) compared to patients without spironolactone. In older patients with HFrEF, spironolactone was more effective regardless of the renal eligibility criteria used [17].

The EMPHASIS-HF trial (n=2737) evaluated the effect of eplerenone (25 to 50 mg) in patients with NYHA class II and EF ≤35%. The patients treated with eplerenone reported a 27% lower risk of hospitalization due to HF and a 23% lower risk of hospitalization for any cause compared to placebo (P<0.001) [18]. The EPHESUS trial on patients with AMI, LVEF ≤40% and clinical signs of HF showed that eplerenone-treated patients (25 to 50 mg) had a 13% lower risk of death from cardiovascular causes or hospitalization for cardiovascular events (P=0.002) and 21 % lower risk of sudden death from cardiac causes (P=0.003) compared to placebo [19].

The summary of treatment benefits of MRA compared to other HF drugs in the treatment of patients with symptomatic HF (stage C) and HFrEF is given in Table 1 [20].

**Table 1:** Comparison of the number needed to treat for mortality reduction in HFrEF patients: MRA versus other drugs ACEI, angiotensin-converting enzyme; ARB, angiotensin-receptor blocker; GDMT, guideline-directed medical therapy; HFrEF, heart failure with reduced ejection fraction; MRA, mineralocorticoid receptor antagonist; NNT, number needed to treat

	MRA	Isosorbide dinitrate and hydralazine	ACEI/ ARB	Beta-blocker
NNT for mortality reduction	6	25	26	9

The overview of guideline recommendations on MRA for patients with HFrEF is given in Table 2 [21,22].

**Table 2:** Overview of guidelines on the recommendation for MRA in HFrEF HF, Heart failure; ACC, American College of Cardiology; AHA, American Heart Association; ESC, European Society of Cardiology; HFrEF, Heart failure with reduced ejection fraction; MRA, mineralocorticoid receptor antagonist; NYHA, New York Heart Association Class 1 (Strong evidence) ** Level A Evidence: High-quality evidence from more than 1 RCT, Meta-analyses of high-quality RCTs, One or more RCTs corroborated by high-quality registry studies

Guideline	Recommendation	Grade
ESC	An MRA is recommended for patients with HFrEF to reduce the risk of HF hospitalization and death	IA
ACC/AHA	In patients with HFrEF and NYHA class II to IV symptoms, an MRA (spironolactone or eplerenone) is recommended to reduce morbidity and mortality, if the estimated glomerular filtration rate is >30 mL/min/1.73 m^2^ and serum potassium is <5.0 mEq/L	IA
	Careful monitoring of potassium, renal function, and diuretic dosing should be performed at initiation and closely monitored thereafter to minimize the risk of hyperkalemia and renal insufficiency	

MRA in heart failure with mildly reduced ejection fraction (HFmrEF)

Enzan et al. studied the prognostic impact of spironolactone treatment on mortality and HF rehospitalization in patients with heart failure with mildly reduced ejection fraction (HFmrEF) in the Japanese Cardiac Registry of HF in Cardiology (JCARE‐CARD) database. At a mean follow-up of 2.2 years, a lower incidence rate of a composite of all-cause death or HF rehospitalization was observed in patients on spironolactone compared to patients without spironolactone (P=0.004) [23].

The TOPCAT sub-group analysis evaluated the relationship between LVEF and the efficacy of spironolactone. The incidence of cardiovascular death was highest in the patients with ejection fraction <50%. Ejection fraction had an impact on the effect of spironolactone on the primary outcome of HF hospitalization/CV death and patients at the lower end of the spectrum of LVEF had significantly greater benefits than the patients on the upper spectrum [24].

An expert consensus document endorsed by ESC recommended that spironolactone may be considered for ambulatory patients (who have no contraindications to the use of spironolactone) with symptomatic HFmrEF to reduce the risk of CV death and HF hospitalization [25].

MRA in heart failure with preserved ejection fraction (HFpEF)

The HF with preserved ejection fraction (HFpEF) refers to the clinical syndrome of heart failure associated with normal or near-normal systolic function. The incidence of HFpEF increases with age and it mostly affects women. HFpEF showed similar morbidity and mortality as those of heart failure with systolic dysfunction [26].

The TOPCAT trial on HFpEF patients reported that 18.6% of patients on spironolactone experienced the primary outcome (composite of death from cardiovascular causes, aborted cardiac arrest, or hospitalization for the management of HF) as compared to 20.4% of patients on placebo (P= 0.14). An increase in the levels of serum creatinine and a doubling of the rate of hyperkalemia was observed in 18.7% of spironolactone-treated patients as compared to 9.1% of patients treated with placebo [27].

MRA initiation in heart failure patients

The efficacy and safety of spironolactone and eplerenone in mortality and hospitalizations are well established from pivotal clinical trials such as RALES, EMPHASIS, and EPHESUS [16,18,19]. However, the timing of the initiation of MRA, especially in patients hospitalized for HF remains uncertain.

Early evidence indicating the impact of timing of initiation of MRA comes from a study by Rossi et al., which evaluated the association between timing of MRA initiation (early, i.e., <30 days’ post discharge vs late, i.e., 30-90 days’ post-discharge) and mortality in patients with HF discharged alive after a first episode of decompensated HF (n=689). A total of 7.1% of patients in the early treatment group, as compared to 13.4% in the late treatment group died after hospitalization at the end of 1-year post-discharge (adjusted HR for mortality for delayed versus early initiation was 1.93 [95% CI: 1.18 to 3.14]). A delay of only one month in administering an MRA implies approximately doubling mortality risk after one year [28].

A posthoc analysis of the EMPHASIS HF trial by Girerd et al. evaluated the timing of initiation of eplerenone in patients with a CV Hospitalization within the past six months. Eplerenone was initiated post-CV hospitalization at a median of 42 days. The event rates were lower in the 42+ days group. A total of 23.6% in the 42+ days met the primary outcome (i.e. composite of CV death and hospitalization for HF) and 28.6% in the 42 days group. The study provides strong evidence of the safety of early initiation of eplerenone after a cardiovascular hospitalization (CVH) [29].

MRAs in acute decompensated heart failure

There is scant data regarding the association between MRA use and outcomes of all-cause mortality and hospital readmission in acute decompensated heart failure (ADHF). This association was evaluated in the Kyoto Congestive Heart Failure (KCHF) registry from Japan [30]. In this registry, 3717 patients were hospitalized for ADHF, and of these, 1678 patients (45.1%) had received MRA at discharge. The matched cohort (n = 1034 in each group) evaluation indicated that the cumulative 1-year incidence of the primary outcome (composite of all-cause death or heart failure hospitalization after discharge) was significantly lower in the MRA use group (28.4%) than in the no MRA use group (33.9%), P=0.003. A 30% reduction in the cumulative 1-year incidence of HF hospitalization was observed in the MRA use group (18.7%) than in the no MRA use group (24.8%), P<0.01. No difference was observed in mortality and all-cause hospitalization. For patients with a left ventricular ejection fraction of 40% or greater, the use of MRA was significantly associated with the primary outcome. MRA use at discharge from ADHF hospitalization was linked to a lower risk of heart failure readmission [30]. The cumulative 1-year outcomes of MRA vs no MRA are given in Table 3.

**Table 3:** Cumulative 1-year outcomes of MRA Vs no MRA MRA, minerolcorticoid receptor antagonist

	MRA	No MRA
The cumulative 1-year incidence of heart failure hospitalization	18.7% HR, 0.70; 95% CI, 0.60-0.86; P < 0.001	24.8%
The cumulative 1-year incidence of the primary outcome	28.4%; HR, 0.81; 95% CI, 0.70-0.93; P = 0.003	33.9%

MRAs and new-onset atrial fibrillation

A meta-analysis and systematic review has shown that MRAs are potential atrial fibrillation (AF) preventive therapy. In MRA-treated patients, a significant overall reduction in AF occurrence was observed as compared to control groups (15.0% versus 32.2%; odds ratio, 0.55; 95% CI, 0.44-0.70 (P<0.001)). The greatest benefit was observed regarding the prevention of recurrent AF episodes (odds ratio, 0.42; 95% CI, 0.31-0.59 (P<0.001)). MRAs appear to be effective in AF prevention, especially recurrent AF episodes [31].

MRAs in worsening renal failure (WRF), hyperkalemia

Aldosterone receptor antagonism influences serum K+ levels by impairing aldosterone-mediated effects on K+ homeostasis in the principal cells of the kidney. Antagonism of the aldosterone receptor, therefore, decreases luminal K+ excretion, thereby increasing the possibility of clinically significant hyperkalemia; when severe, hyperkalemia may precipitate cardiomyocyte membrane potential destabilization and unstable ventricular arrhythmias [32].

Although no death attributable to hyperkalemia was reported in the RALES, EPHESUS, or EMPHASIS-HF studies, the subsequent pharmacovigilance studies published after the RALES study found an increase in the reporting of hyperkalemia in CHF patients treated with spironolactone [33].

The EMPHASIS-HF study reported an increased incidence of hyperkalemia among patients receiving eplerenone. A serum potassium level above 5.5 mmol/L was reported in 11.8% of the eplerenone group and 7.2% in the placebo group (P<0.001). Moreover, a serum potassium level above 6.0 mmol/L was reported in 2.5% in the eplerenone group and 1.9% in the placebo group (P=0.29). Although 8% of HF patients on eplerenone develop hyperkalemia, only 1% withdrew due to an adverse event [18].

The association with hyperkalemia and worsening renal function (WRF) with eplerenone was assessed in a post hoc analysis of the EMPHASIS- HF study. In this post hoc analysis, all 2737 patients with HFrEF were included. The addition of eplerenone was associated with an increased incidence of WRF and hyperkalemia. Although eplerenone induced a significant rise in K+ levels (P<0.004), the rise was modest [34]. These adverse outcomes did not negate the major survival benefit of eplerenone when systematic monitoring of electrolyte and kidney function was performed, and eplerenone doses were adjusted based on renal function and potassium concentration [34].

The management of hyperkalemia and hypokalemia and spironolactone and eplerenone dose adjustment proposal is given in Table 4 and Table 5, respectively [35].

**Table 4:** Management of hyperkalemia and hypokalemia ACEI, angiotensin-converting enzyme inhibitors; ARBs, angiotensin receptor blockers; MRA, mineralocorticoid receptor antagonists; NSAIDs, non-steroidal anti-inflammatory drugs; RAASi, renin-angiotensin-aldosterone system inhibitors

Hyperkalemia>5.5mmol/L
·Assess the possibility of hemolysis.
·Initiate diuretics or increase its dose (if necessary)
·Eliminate K+ supplements, NSAIDs and decrease potassium-rich foods
·Replace ACEI / ARBs by sacubitril-valsartan
·Adapt MRA dose if necessary
·Consider a K+ binder (do not stop RAASi)
Hypokalemia <4 mmol/L
·Stop thiazides; prefer loop diuretics for relief of congestion
·Initiate MRA (or increase the dose if already taking one)
·Increase ACE inhibitors / ARBs dose to guideline-recommended targets
·Monitor K+ and creatinine

**Table 5:** Spironolactone and eplerenone dose adjustment proposal eGFR, estimated glomeruli filtration rate; MRA, mineralocorticoid receptor antagonists

Serum potassium (mmol/l)	Dose adjustment
	Baseline eGFR ≥50ml/min/1.73m^2^ spironolactone dose =25 mg/day or eplerenone 50 mg/day
	Baseline eGFR 30-49 ml/min/1.73m^2^ spironolactone dose =25 mg every other day or eplerenone 25 mg/day
<4.0	Increase dose: If the spironolactone dose is 25 mg/day, then increase to 50 mg/day, or if the eplerenone dose is 50 mg/day then increase to eplerenone 100 mg/day. If the spironolactone dose is 25mg every other day, then increase to 25 mg/day, or if the eplerenone dose is 25 mg/day then increase to 50 mg/day
4.0-5.4	No adjustment recommended
5.5 -5.9	Decrease dose. If the spironolactone dose =50mg/day, then decrease the dose to 25 mg/day, or if the eplerenone dose is 100 mg/day, then decrease the dose to 50 mg/day. If the spironolactone dose =25mg/day, then decrease the dose to 25 mg every other day, or if eplerenone dose is 50 mg/day, then decrease the dose to 25 mg/day. If the spironolactone dose 25 mg every other day, then interrupt treatment and reassess potassium within 1 week or if the eplerenone dose = 25mg/day, interrupt treatment and reassess potassium within 1 week
>6.0	Stop MRA treatment and reassess potassium levels after 1 week. When potassium levels are <6.0 mmol/L, initiate a K+ binder and reintroduce MRA. Stop MRA treatment in any case if eGFR≤30 ml/min/1.73m^2^ and reintroduce upon clinical decision i.e. upon renal functional improvement and K+ stabilization

Expert opinion recommendations

A panel of expert cardiologists from India convened to develop this expert opinion document to guide physicians on the optimal use of MRAs in HF patients. Clinical practice-based recommendations are provided in Table 6.

**Table 6:** Clinical practice-based recommendations ACEi, angiotensin-converting enzyme inhibitors; AMI, acute myocardial infarction; ARNi, angiotensin receptor neprilysin inhibitors; BNP, brain natriuretic peptide; eFGR, estimated glomeruli filtration rate; HF, heart failure; HFmrEF, heart failure with mid-range ejection fraction; HFpEF, heart failure with preserved ejection fraction; HFrEF, heart failure with reduced ejection fraction; MRA, mineralocorticoid receptor antagonists; RAASi, renin-angiotensin-aldosterone system inhibitors; TOPCAT,  treatment of preserved cardiac function heart failure with an aldosterone antagonist trial

Clinical practice-based recommendations
· In HFrEF, MRA may provide cardiovascular protection beyond its diuretic and potassium-sparing properties.
· In HFrEF, Spironolactone has specific neurohormonal activity on the RAAS system, which includes a reduction in BNP and an increase in AT II and aldosterone due to feedback. It should be emphasized that despite treatment with ACEi, there may be elevated angiotensin 2, which stimulates aldosterone levels via an escape phenomenon.
· In HFrEF, suppression of collagen turnover changes and reduction in markers of cardiac fibrosis may be one of the extrarenal mechanisms of the beneficial effect of spironolactone and eplerenone.
· In HFrEF patients who are symptomatic despite RAASi/ARNI/Beta-blockers and with Serum K^+^ < 5.0 meq/L and eGFR > 30 ml/min/m^2^, MRAs such as spironolactone or eplerenone are recommended to reduce the risk of mortality and hospitalization.
· Earlier introduction of eplerenone in the pre-discharge hospital setting, especially in post-AMI patients without contraindications, is likely beneficial. In chronic HF and hospitalized HF, it may be beneficial to initiate MRA early after ACEi and beta blockers. Additionally, it must be emphasized that eplerenone and spironolactone should be initiated only after ACE-/ARBs and beta blockers.
· In HFrEF patients, spironolactone may reduce the incidence of ventricular premature contractions and ventricular tachycardia.
· There are no head-to-head comparisons of eplerenone and spironolactone in HF. However, eplerenone may benefit the ischemic subset of HFrEF and may be more suitable for male patients who develop breast tenderness or gynecomastia. Spironolactone may be preferred in patients from poor socioeconomic groups.
· In HFpEF patients with specific selection criteria like phenogroup 3 from the TOPCAT sub-study (and with EF >45%, elevated BNP levels or HF admission within 1 year, estimated glomerular filtration rate >30 mL/min, creatinine <2.5 mg/dL, potassium <5.0 mEq/L), aldosterone receptor antagonists such as spironolactone or eplerenone may be considered to decrease hospitalizations.
· In HFmrEF patients who are symptomatic despite RAASi and beta-blockers and who have serum K^+^ < 5.0 meq/l and with eGFR> 30 ml/min/m^2^, Spironolactone may be considered.
· In HF patients on MRAs, routine testing for renal function and electrolytes (particularly K^+^) is recommended.
· Low-dose MRA at initiation (25 mg of eplerenone or spironolactone) should be considered. MRAs should be up-titrated after 4–8 weeks.
· Check serum electrolytes and serum creatinine at 1 and 4 weeks after starting/increasing dose and at 8 and 12 weeks; 6, 9, and 12 months; 4 months thereafter. Repeat electrolyte estimation is recommended for confirmation of initial high levels of serum K^+^ to avoid lab errors. Alternative causes of hyperkalemia should be ruled out.
· If K^+ ^rises above 5.5 mmol/L or creatinine, rises to 2.5 mg/dL, or eGFR < 30 ml/min/m^2^, the dose of MRA should be reduced to half, and blood chemistry monitored closely.
· If K^+^ rises to >6.0 mmol/L or creatinine to 3.5 mg/dL eGFR <20 ml/min/1.73 m^2, ^stop MRA and seek specialist advice.
· There are no head-to-head comparisons of eplerenone and spironolactone in HF and no evidence that either is more beneficial in HFrEF with Diabetes.
· In diabetic patients, serum electrolytes should be monitored vigilantly, irrespective of renal function.

## Conclusions

The reason for the underutilization of MRA is multifactorial. The chief limiting factor in the mind of the treating doctor is the fear of hyperkalemia associated with using MRA in HF patients. Secondly, an apprehension that the patient might not comply with serum K+ monitoring. In a multispecialty setup, the prescription of MRA is often considered far down in the algorithm of HF management and is missed out in the discharge medications.

This expert group opines that there is an urgent need to highlight the pathophysiological role and mechanism of action of MRAs as well as to educate physicians involved in HF management about the need and benefit of safe use of MRAs in HF.
